# Modulation of Visual Contrast Sensitivity with tRNS across the Visual System, Evidence from Stimulation and Simulation

**DOI:** 10.1523/ENEURO.0177-22.2023

**Published:** 2023-06-12

**Authors:** Weronika Potok, Alain Post, Valeriia Beliaeva, Marc Bächinger, Antonino Mario Cassarà, Esra Neufeld, Rafael Polania, Daniel Kiper, Nicole Wenderoth

**Affiliations:** 1 Neural Control of Movement Lab, Department of Health Sciences and Technology, ETH Zürich, Zurich 8093, Switzerland; 2 Neuroscience Center Zurich (ZNZ), University of Zurich, ETH Zurich, University and Balgrist Hospital Zurich, Zurich 8057, Switzerland; 3 Decision Neuroscience Lab, Department of Health Sciences and Technology, ETH Zürich, Zurich 8057, Switzerland; 4 Foundation for Research on Information Technologies in Society (IT’IS), Zurich 8004, Switzerland; 5 Life Science Zurich Learning Center, Zurich 8057, Switzerland; 6 Future Health Technologies, Singapore-ETH Centre, Campus for Research Excellence And Technological Enterprise (CREATE), 138602, Singapore

**Keywords:** contrast detection, E-field modeling, neuromodulation, sensory system, stochastic resonance, transcranial electrical stimulation

## Abstract

Transcranial random noise stimulation (tRNS) has been shown to significantly improve visual perception. Previous studies demonstrated that tRNS delivered over cortical areas acutely enhances visual contrast detection of weak stimuli. However, it is currently unknown whether tRNS-induced signal enhancement could be achieved within different neural substrates along the retino-cortical pathway. In three experimental sessions, we tested whether tRNS applied to the primary visual cortex (V1) and/or to the retina improves visual contrast detection. We first measured visual contrast detection threshold (VCT; *N* = 24, 16 females) during tRNS delivery separately over V1 and over the retina, determined the optimal tRNS intensities for each individual (ind-tRNS), and retested the effects of ind-tRNS within the sessions. We further investigated whether we could reproduce the ind-tRNS-induced modulation on a different session (*N* = 19, 14 females). Finally, we tested whether the simultaneous application of ind-tRNS to the retina and V1 causes additive effects. Moreover, we present detailed simulations of the induced electric field across the visual system. We found that at the group level tRNS decreases VCT compared with baseline when delivered to the V1. Beneficial effects of ind-tRNS could be replicated when retested within the same experimental session but not when retested in a separate session. Applying tRNS to the retina did not cause a systematic reduction of VCT, regardless of whether the individually optimized intensity was considered or not. We also did not observe consistent additive effects of V1 and retina stimulation. Our findings demonstrate significant tRNS-induced modulation of visual contrast processing in V1 but not in the retina.

## Visual Abstract

**Figure:**
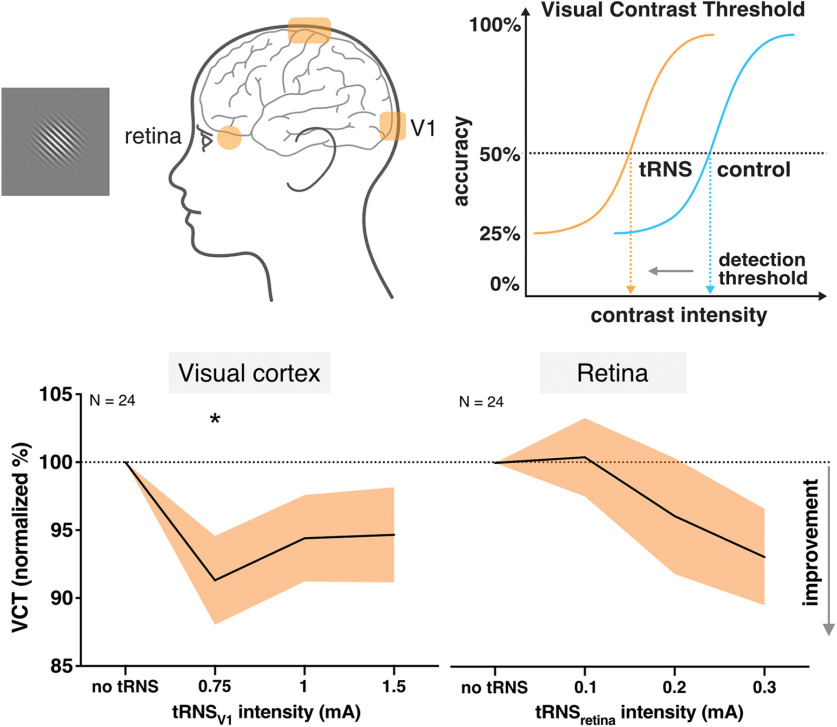

## Significance Statement

Our findings confirm previous evidence showing acute online benefits of transcranial random noise stimulation (tRNS) of primary visual cortex (V1) on visual contrast detection in accordance with the stochastic resonance (SR) phenomenon. We further extend it, demonstrating that the optimal tRNS intensity varies among participants, but when individually tailored it can improve visual processing when re-tested within the experimental session. The tRNS-induced enhancement in visual sensitivity was observed for cortical contrast processing, but stimulation of the retina did not lead to systematic effects.

## Introduction

Transcranial random noise stimulation (tRNS) has been shown to significantly improve visual perception (for review, see Potok et al., 2022) when applied to visual cortex. Such performance improvements can manifest as both after-effects of visual training combined with tRNS in healthy participants (Fertonani et al., 2011; Pirulli et al., 2013; Contemori et al., 2019; Herpich et al., 2019) and patients with visual deficits (Camilleri et al., 2016; Herpich et al., 2019; Moret et al., 2018), or as acute effects during tRNS (van der Groen and Wenderoth, 2016; Ghin et al., 2018; van der Groen et al., 2018, 2019; Battaglini et al., 2019, 2020; Pavan et al., 2019). Studies exploring the acute effects of tRNS on visual processing have shown that noise stimulation of the primary visual cortex (V1) improves stimulus contrast detection, particularly, when visual stimuli are presented with near-threshold intensity (van der Groen and Wenderoth, 2016; Battaglini et al., 2019). Accordingly, studies investigating the acute effect of tRNS on visual detection performance postulated that the stochastic resonance (SR) phenomenon underlies the noise-induced signal enhancement (van der Groen and Wenderoth, 2016; van der Groen et al., 2018, 2019; Battaglini et al., 2019, 2020; Pavan et al., 2019). SR describes the phenomenon where an optimally adjusted additive random noise enhances the detection probability of weak, subthreshold signals in nonlinear systems (Moss et al., 2004; McDonnell and Abbott, 2009). One important feature indicative of the SR phenomenon is that noise benefits are a function of noise intensity and exhibit an inverted U-shape relationship. Thus, while the optimal level of noise benefits performance, excessive noise is detrimental (van der Groen and Wenderoth, 2016; van der Groen et al., 2018; Pavan et al., 2019).

What remains unknown is whether tRNS-induced signal enhancement, and related contrast sensitivity benefits, could be achieved at the retinal level. Modelling studies suggest noise benefits in retinal ganglion cells (Patel and Kosko, 2005) induced by both visual (Ghosh et al., 2009) and electrical noise (Wu et al., 2017). Moreover, previous research has suggested that the retina is susceptible to 8- to 20-Hz transcranial alternating current stimulation (tACS; Schutter and Hortensius, 2010; Kar and Krekelberg, 2012), which induces phosphenes even if the stimulation electrodes are placed over distal locations of the scalp (Laakso and Hirata, 2013; for review, see Schutter, 2016). Interestingly, improvement in vision was reported after repetitive transorbital alternating current stimulation at 5–30 Hz over the retina of patients with optic neuropathy or after optic nerve lesions (Gall et al., 2010, 2011; Fedorov et al., 2011; Sabel et al., 2011). They suggested that observed improvements were mediated by increased neuronal synchronization of residual structures and higher cortical areas within the visual system (Sabel et al., 2011). The retina and the optic nerve are interesting targets because they can be reliably reached even with low transcranial electrical stimulation (tES) intensities since the eyeball is an excellent conductor (Haberbosch et al., 2019). However, it remains unknown whether noise benefits resulting from tRNS can be induced at different levels of the retino-cortical processing pathway.

In this preregistered study, we investigated the effects of tRNS stimulation of the retina, primary visual cortex (V1) or both on visual detection performance.

## Materials and Methods

This study was preregistered on the Open Science Framework platform (https://osf.io/gacjw). The only difference to preregistered original plan concerns the included sample population. We stated that only participants who completed all three sessions will be included in our study. During data collection not all the individuals who completed the first and second sessions participated in the third session, because of the COVID-19 pandemic (Bikson et al., 2020). Nevertheless, we decided to keep all the data collected in sessions 1 and 2 (*N* = 24) despite dropouts and lower sample size in session 3 (*N* = 19; see below, Participants).

### Participants

Only individuals with no identified contraindications for participation according to established brain stimulation exclusion criteria (Wassermann, 1998; Rossi et al., 2009) were recruited for the study. All study participants provided written informed consent before the beginning of each experimental session. Upon study conclusion, they were debriefed and financially compensated for their time and effort. All research procedures were approved by the Cantonal Ethics Committee Zurich (BASEC Nr. 2018-01078) and were performed in accordance with the Helsinki Declaration of the World Medical Association (2013 WMA Declaration of Helsinki) and guidelines for noninvasive brain stimulation (NIBS) research through the COVID-19 pandemic (Bikson et al., 2020).

**Figure 1.**
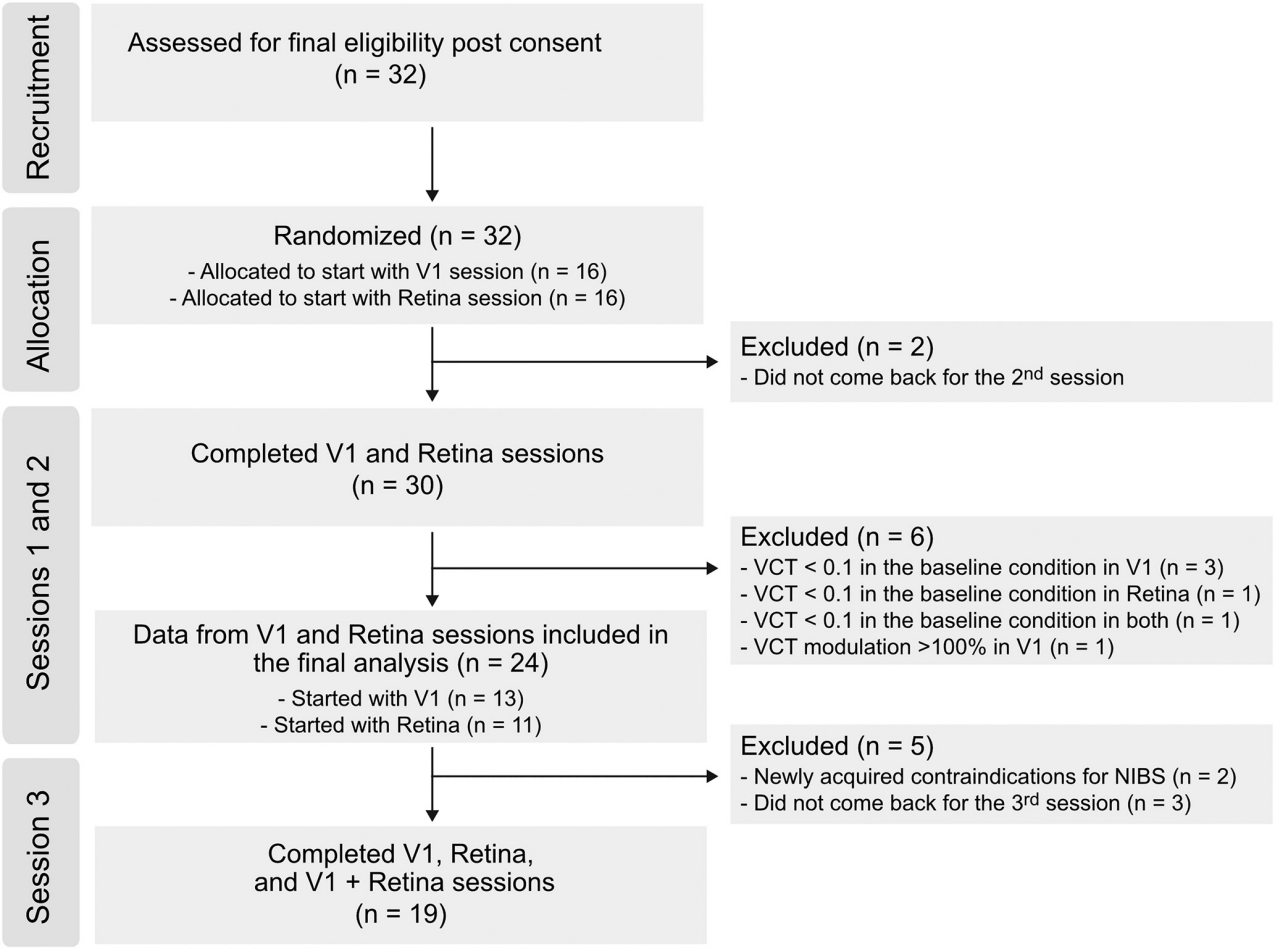
Flow diagram of the data collection progress through the phases of the study.

The required sample size was estimated using an a priori power analysis (G*Power version 3.1; Faul et al., 2007). Based on previous finding from van der Groen and Wenderoth (2016) we expected the effect of maximum contrast sensitivity improvement to correspond to Cohen’s *d* = 0.77. The power analysis revealed that fourteen participants should be included in an experiment to detect an effect of tRNS on contrast detection with repeated measures (rm)ANOVA (four levels of stimulation condition), α = 0.05, and 90% power, assuming the correlations among repeated measures = 0.5. However, there was no prior data available to investigate whether applying tRNS to two separate neural structures can cause additive effects. Therefore, we include more participants to ensure sufficient power. Moreover, this estimation hinges on the assumption that approx. 80% of the participants exhibit a behavioral response to tRNS (as indicated by van Der Groen and Wenderoth, 2016). Thus, we collected data until *N* = 20 responders have been included. Responders were defined as individuals who exhibited improved detection in at least one tRNS condition in V1 and retina stimulation. Visual contrast detection is potentially prone to floor effects if the contrast detected at baseline approaches the technical limits of the setup. We decided to exclude participants that were exceptionally good in the visual task and present visual contrast threshold below 0.1 in the no tRNS baseline condition (Michelson contrast, see below, Visual stimuli). We also excluded individuals with exceptional contrast threshold modulation in tRNS trials with respect to no tRNS trials (>100%) to avoid accidental results, e.g., because of participants responding without paying attention to the task. From the initially recruited sample of 32 participants, we excluded eight individuals [five participants had a contrast threshold below 0.1 in the baseline condition of one of the stimulation sessions (V1 or retina), one participant revealed exceptional contrast threshold modulation (>100%), two participants did not come back for the second session]. The final sample consisted of 24 healthy volunteers (16 females, 8 males; 24.4 ± 4.1, age range: 21–38) with normal or corrected-to-normal vision (see Fig. 1). A total number of 24 individuals participated in the first two sessions, with tRNS over V1 and tRNS over the retina (counterbalanced in order). Because of the COVID-19 pandemic, we were forced to stop data collection for several months (Bikson et al., 2020). After returning to the lab, five participants dropped-out from the initial sample [two had newly acquired contraindications for noninvasive brain stimulation (NIBS) and three were not able to participate]. A total of 19 healthy volunteers (14 females, 5 males; 25.5 ± 5.2, age range: 21–39) were included into third session (tRNS over V1 and retina; see Fig. 1).

### General study design

**Figure 2.**
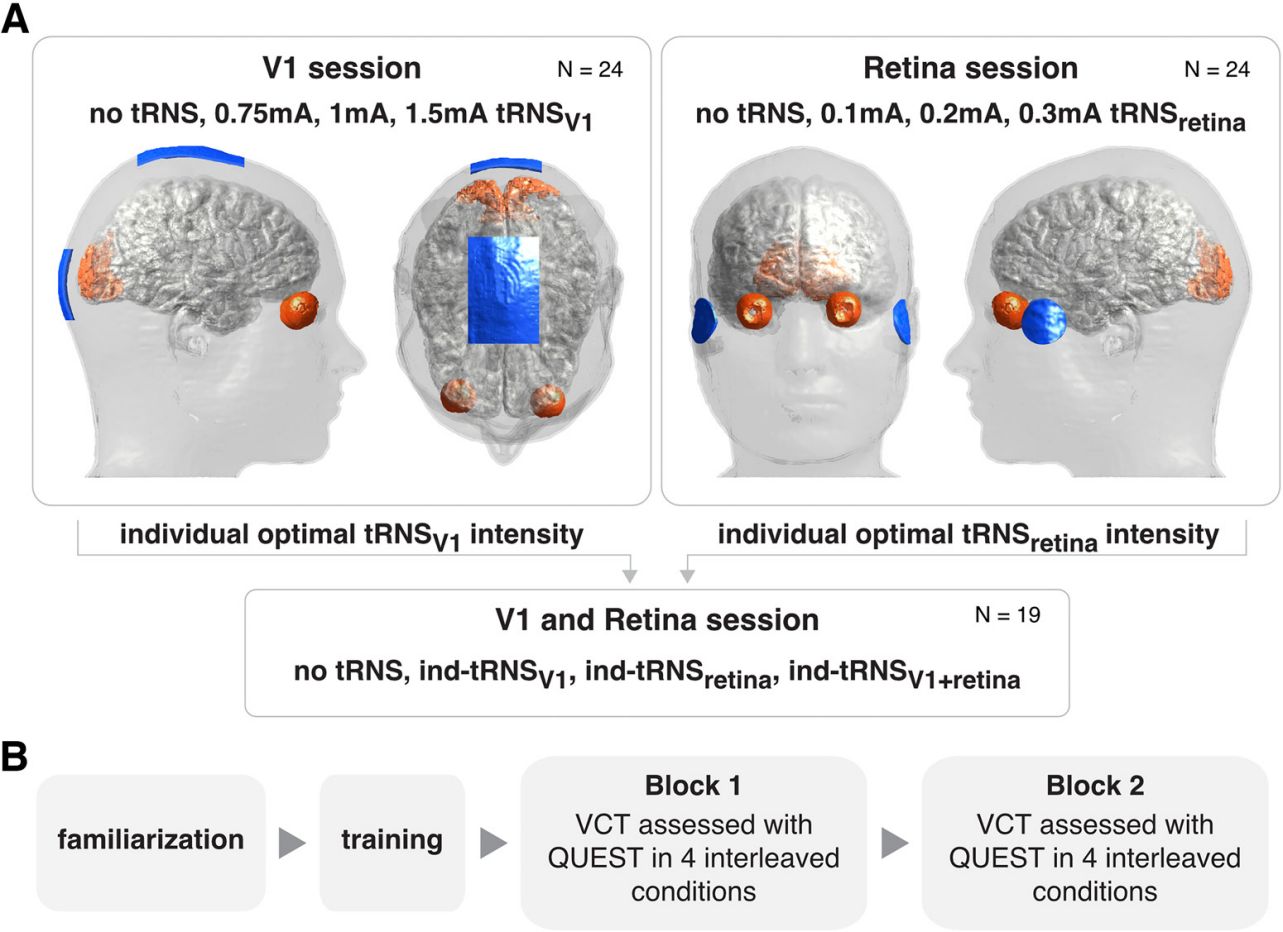
***A***, Experimental design, stimulation parameters, electrodes montage (blue), and masks of the main targeted regions of interest (orange). First, participants completed experimental sessions in which they received tRNS over V1 or retina (counterbalanced in order) and where the optimal individual tRNS intensity (ind-tRNS) was defined based on the behavioral performance. Next, the ind-tRNS was applied on the third session separately or simultaneously over V1 and retina in a randomized order. ***B***, The order of measurements within each session. Each experimental session consisted of a familiarization protocol, followed by task training and two independent visual contrast threshold (VCT) assessments in four interleaved tRNS condition (as specified in ***A***).

To evaluate the influence of tRNS on visual contrast detection, we performed a series of three experimental sessions in which we delivered tRNS over different levels of the visual system, namely, V1, retina, or simultaneously over both V1 and retina (V1+Retina), during visual task performance (see Fig. 2*A*). In each experiment, tRNS at low, medium and high intensity and a control no tRNS condition were interleaved in a random order (see below, tRNS characteristics).

The order of experimental sessions for V1 and retina stimulation were counterbalanced across participants (13 participants started with V1 and 11 with retina stimulation). These experimental sessions took place on different days which were on average two weeks apart. Because of COVID-19 restrictions, the third session had to be delayed by five months on average.

Our main outcome parameter in all experimental sessions was a threshold of visual contrast detection (VCT) that was determined for each of the different tRNS conditions. VCT was independently estimated twice, in two separate blocks within each session (see Fig. 2*B*). During the first two sessions we determined the individual optimal tRNS intensity (defined as the intensity causing the lowest VCT, i.e., biggest improvement in contrast sensitivity) for each participant in the V1 session (ind-tRNS_V1_) and the retina session (ind-tRNS_retina_). In the third session we then applied ind-tRNS_V1_ and ind-tRNS_retina_ to investigate the effect on VCT when V1 and retina are stimulated simultaneously.

#### Visual stimuli

**Figure 3.**
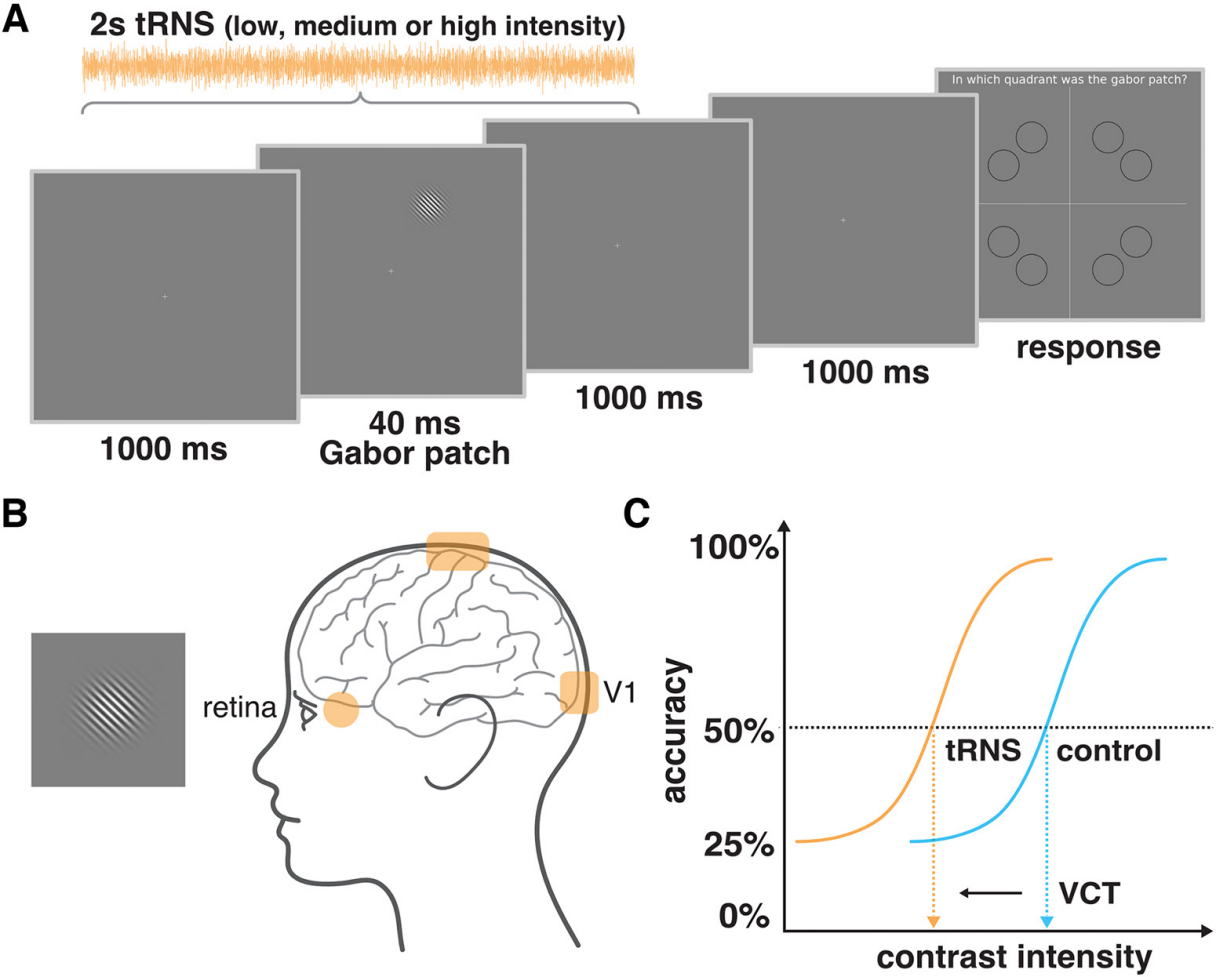
Experimental design. ***A***, Example trial of four-alternative forced choice task measuring visual contrast detection threshold (VCT). tRNS started 20 ms after trial onset and was maintained for 2 s. ***B***, Exemplary Gabor patch stimulus to be detected during the visual task and tRNS electrodes montage targeting V1 (rectangle) or retina (round, only the left side is shown but electrodes were mounted bilaterally). ***C***, Example of dose–response psychometric curves and the estimation of VCT for the 50% detection accuracy level. We hypothesize that the VCT will be lower (indicating better contrast detection performance of the participant) in one of the tRNS conditions (orange) than in the no tRNS control condition (blue).

All experiments took place in a dark and quiet room, ensuring similar lighting conditions for all participants. Participants sat comfortably, 0.85 m away from a screen, with their head supported by a chinrest. Visual stimuli, i.e., Gabor patches, were generated with MATLAB (MATLAB 2020a, MathWorks) using a function in the Psychtoolbox extension that defines the stimulus intensity with Michelson contrast (Brainard, 1997; Kleiner et al., 2007; Pelli, 1997) and displayed on a CRT computer screen (Sony CPD-G420). The screen was characterized by a resolution of 1280 × 1024 pixels, refresh rate of 85 Hz, linearized contrast, and a luminance of 35 cd/m^2^ (measured with J17 LumaColor Photometer, Tektronix). The target visual stimuli were presented on a uniform gray background in the form of a Gabor patch, a pattern of sinusoidal luminance grating displayed within a Gaussian envelope (full width at half maximum of 2.8 cm, i.e., 1° 53' visual angle, with 7.3 cm, i.e., 4° 55' presentation radius from the fixation cross, staying within the central vision, i.e., <8° radius; Strasburger et al., 2011; Younis et al., 2019). The Gabor patch pattern consisted of 16 cycles with one cycle made up of one white and one black bars (grating spatial frequency of 8 cycles/deg). Stimuli were oriented at 45° tilted to the left from the vertical axis (see Fig. 3*B*), since it was shown that tRNS enhances detection of low contrast Gabor patches especially for nonvertical stimuli of high spatial frequency (Battaglini et al., 2020).

#### Four-alternative forced choice visual detection task

In all three experiments participants performed a four-alternative forced choice (4-AFC) visual task, designed to assess an individual VCT, separately for each tRNS condition. A 4-AFC protocol was shown to be more efficient for threshold estimation than commonly used 2-AFC (Jäkel and Wichmann, 2006). Participants were instructed to fixate their gaze on a cross in the center of the screen. In the middle of each 2.04-s trial, a Gabor patch was presented for 40 ms in one of the eight locations (see Fig. 3*A*). To account for potential differences in the extent to which tRNS affects different retinotopic coordinates and to avoid a spatial detection bias, the visual stimuli were presented pseudo-randomly and appeared the same number of times (20) in each of the eight locations on the screen within each experimental block (van der Groen and Wenderoth, 2016). The possible locations were set on noncardinal axes, as the detection performance for stimuli presented in this way is less affected (i.e., less variable) than when stimuli are positioned on the cardinal axes (Cameron et al., 2002). The trial was followed by 1s presentation of fixation cross after which the “response screen” appeared. Participants’ task was to decide in which quadrant of the screen the visual stimulus appeared and indicate its location on a keyboard. The timing of the response period was self-paced and not limited. Participants completed a short training session (10 trials), with the stimulus presented always at high contrast (0.5; for visual contrast intensity range of minimum 0 and maximum 1), to ensure that they understand the task (Fig. 2*B*).

During the main experiment, VCT was estimated using the QUEST staircase maximum likelihood procedure (Watson and Pelli, 1983) implemented in the Psychophysics Toolbox in MATLAB (Brainard, 1997; Pelli, 1997; Kleiner et al., 2007). The thresholding procedure starts with a presentation of the visual stimulus displayed with 0.5 contrast intensity (Michelson contrast, for visual contrast intensity ranging 0–1; note that the stimuli were displayed for just 40 ms). When participants answer correctly QUEST lowers the presented contrast intensity, when participants answer incorrectly QUEST increases the presented contrast. The estimated stimulus contrast is adjusted to yield 50% detection accuracy (i.e., detection threshold criterion; see Fig. 3*C*). Note, that for 4-AFC task 25% accuracy corresponds to a chance level. The remaining parameters used in the QUEST staircase procedure included: steepness of the psychometric function, β = 3; fraction of trials on which the observer presses blindly, δ = 0.01; chance level of response, γ = 0.25; step size of internal table grain = 0.001; intensity difference between the largest and smallest stimulus intensity, range = 1. VCT was assessed across 40 trials per tRNS condition (40 trials × four conditions × two blocks; total number of 320 trials per experimental session).

#### tRNS characteristics

In tRNS trials, high-frequency tRNS (hf-tRNS, 100–640 Hz) with no offset was delivered. The probability function of random current intensities followed a Gaussian distribution with 99% of the values lying between the peak-to-peak amplitude (Potok et al., 2022). Stimulation started 20 ms after trial onset and was maintained for 2 s (Fig. 3*A*). Subsequently a fixation cross was displayed for 1s, followed by the self-paced response time. On every session, a new tRNS waveform was created in each trial within MATLAB (MATLAB 2020a, MathWorks) and sent to a battery-driven electrical stimulator (DC-Stimulator PLUS, NeuroConn GmbH), operated in REMOTE mode, via a National Instruments I/O device (USB-6343 X series, National Instruments). The active tRNS conditions and no tRNS control condition were interleaved and presented in random order. Timing of the stimuli presentation, remote control of the tRNS stimulator, and behavioral data recording were synchronized via MATLAB (MATLAB 2020a, MathWorks) installed on a PC (HP EliteDesk 800 G1) running Windows (Windows 7, Microsoft) as an operating system. The impedance between the electrodes was monitored and kept below 15 kΩ.

Since we used a very brief stimulation time (2 s only), fade in/out periods were not possible (Potok et al., 2021). Accordingly, some participants were able to distinguish the stimulation conditions (see Results). We accounted for this possible bias using a control measure and analysis of the potential transcutaneous sensations. In each session, before the start of the main experiment, participants were familiarized with tRNS and we assessed the detectability of potential cutaneous sensations (Fig. 2*B*). The detection task consisted of 20 trials. Participants received either 5s tRNS (0.75-, 1-, and 1.5-mA peak-to-baseline amplitude tRNS in V1 session; 0.1-, 0.2-, and 0.3-mA peak-to-baseline amplitude tRNS in the retina session; or ind-tRNS_V1_, ind-tRNS_retina_, ind-tRNS_V1+retina_ in V1+Retina session) or no tRNS. Their task after each trial was to indicate on a keyboard whether they felt a sensation underneath the tRNS electrodes. The determined detection accuracy (hit rates, HR, defined as the proportion of trials in which a stimulation is present and the participant correctly responds to it) of the cutaneous sensation induced by tRNS served as a control to estimate whether transcutaneous effects of the stimulation might have confounded the experimental outcomes (Potok et al., 2021). In the control analysis we averaged the HR for tactile detection across tRNS stimulation conditions (separately for tRNS_V1_, tRNS_retina_, and tRNS_V1+retina_) and used the mean HR as a covariate (see below, Statistical analysis).

##### V1 session: testing the effect of no, low-, medium-, or high-intensity tRNS targeting V1 on visual detection performance

In the V1 session, we asked whether tRNS over V1 modulates VCT. To target V1 we used an electrode montage that was previously shown to be suitable for V1 stimulation (van der Groen and Wenderoth, 2016; Herpich et al., 2019). One tRNS 5x5cm rubber electrode was placed over the occipital region (3 cm above inion, Oz in the 10–20 EEG system) and one 5 × 7-cm rubber electrode over the vertex (Cz in the 10–20 EEG system). Electroconductive gel was applied to the contact side of the rubber electrodes (NeuroConn GmbH) to reduce skin impedance.

tRNS was delivered with 0.75 mA (low), 1 mA (medium), and 1.5 mA (high) amplitude (peak-to-baseline), resulting in maximum current density of 60 $\frac{\mu A}{cm2}$, which is below the safety limits of 167 $\frac{\mu A}{cm2}$ for transcranial electrical stimulation (Fertonani et al., 2015). tRNS power, corresponding to the variance of the electrical noise intensities distribution, was 0.109, 0.194, and 0.436mA^2^ in the 0.75, 1, and 1.5 mA condition, respectively (Potok et al., 2022).

##### Retina session: testing the effect of no, low-, medium-, or high-intensity tRNS targeting the retina on visual detection performance

To further explore the influence of electrical random noise on visual processing we delivered tRNS over the retina during a visual contrast detection task. To stimulate the retina, face skin-friendly self-adhesive round electrodes with a diameter of 32 mm (TENS-EMS pads Axion GmbH) were placed on the sphenoid bones of the right and left temples (F9 and F10 in the 10–20 EEG system). Electroconductive gel was applied to the contact side of each electrode to additionally reduce skin impedance.

Dose–response effects were assessed with VCT during tRNS applied with the intensity of 0.1-mA (low), 0.2-mA (medium), and 0.3-mA (high) amplitude (peak-to-baseline), resulting in a maximum current density of 29.3 $\frac{\mu A}{cm2}$, which is well below the safety limits for transcranial electrical stimulation (Fertonani et al., 2015). tRNS power, corresponding to the variance of the electrical noise intensities distribution, was 0.002, 0.008, and 0.017 mA^2^ in the 0.1-, 0.2-, and 0.3-mA condition, respectively (Potok et al., 2022). The choice of used intensities was influenced by three aspects: (1) previous literature, (2) pilot experiments, and (3) considering the discomfort induced by transcranial electrical stimulation (tES). The selected intensities are commonly used in transorbital alternating current stimulation studies that have reported stimulation induced effects (Gall et al., 2010, 2011; Fedorov et al., 2011; Sabel et al., 2011). Furthermore, we had performed a pilot experiment (*N* = 30) to assess a flickering threshold when low-frequency tRNS was used (0.1–100 Hz). We found that flickering was perceived for a mean intensity of 0.146 ± 0.08 mA (peak-to-baseline) suggesting that the stimulation intensities chosen in this experiment should be suitable to reach and effectively stimulate the retina. Interestingly, perceived flickering during low-frequency tRNS suggests a suprathreshold influence of the stimulation, in contrast to stimulation levels usually induced in the brain using tES, i.e., potentially more effective. Note also, that the sphenoid bones are much thinner than the back of the skull. Our pilot experiment further revealed that flickering was induced by low-frequency tRNS but not by high-frequency tRNS (as used in the main experiments), thus the visual task remained unaffected by flickering sensation during hf-tRNS sessions. Finally, we designed our stimulation conditions considering the feasibility of delivering tES to the face of the participants. The skin around the eyes is quite sensitive for most of the people and increasing the intensity for retinal stimulation could be painful for the participants. Based on feedback provided by the pilot participants we found that higher intensities (above the ones used here) resulted in discomfort induced by tES applied to the temples. Importantly, the discomfort because of tES could then additionally influence the task performance.

##### V1+Retina session: testing the additive effect of simultaneously applying tRNS to V1 and the retina on visual detection performance

The final experimental session aimed to investigate potential additive effects of delivering electrical random noise simultaneously to V1 and the retina on visual contrast sensitivity.

In this session, we combined the electrodes montages over V1 and the retina and applied tRNS with individual optimal intensities as determined in the first two experimental sessions (ind-tRNS_V1_ and ind-tRNS_retina_, corresponding to the intensity causing the lowest VCT, i.e., biggest improvement in contrast sensitivity during V1 and retina sessions; see above, General study design).

In the V1+Retina session, we compared the VCT in four conditions: (1) tRNS over V1 at its optimal intensity (ind-tRNS_V1_), (2) tRNS over retina at its optimal intensity (ind-tRNS_retina_), (3) simultaneous tRNS over V1 and the retina at their respective optimal intensities (ind-tRNS_V1+retina_), and (4) no tRNS. All conditions were interleaved and presented in a randomized order.

### Electric field modeling

Electric field modeling was performed for all experimental conditions after the experiments were conducted, to better understand the obtained results. The induced E-field was assessed within several areas along the visual pathway, namely, the retina, optic nerve, optic chasm, optic tract, posterior thalamus (including lateral geniculate nucleus, LGN), and V1.

To assess the exposure related to the stimulation over the visual system under different electrode configurations, a computational model, which replicated the intervention, was created using Sim4Life (ZMT Zurich Med Tech AG) platform for computational life-sciences investigations. To execute the electromagnetic (EM) simulations, we selected the detailed anatomic head model (Fig. 2*A*), i.e., MIDA (Iacono et al., 2015), which distinguishes 117 anatomic regions, including different parts of the visual system: retina, optic nerve, optic chiasm, and optic tract. Additionally, we identified two other areas of the visual path. First, the LGN (obtained as the posterior portion of the thalamus), and secondly, the V1 region that was determined through co-registration of the MIDA model with the open-access Brainnetome atlas (Fan et al., 2016). In the EM simulations, the regions were grouped into 37 tissue classes and electric conductivity values were assigned according to the IT’IS Low Frequency Database V4.1 (IT’ISFoundation, 2022). Code accessible at: https://github.com/vabelyaeva/modeling_visual_system.

To replicate the experimental setup, we generated four electrodes that were positioned on the skin of the MIDA model in accordance with the EEG 10–20 system (Fig. 2*A*). The first two electrodes that were applied for retina stimulation, were designed as cylinders (radius = 16 mm) and placed at the F9 and F10 locations. The second pair of electrodes aimed at targeting of the V1 region had rectangular shapes and were positioned at Cz (5 × 7 cm) and Oz (5 × 5 cm). To evaluate the current and to normalize the E-field distribution to the total current, one electrode in each configuration was surrounded with a sensor box (see above, tRNS characteristics).

The EM simulations were performed using Sim4Life’s rectilinear version of the “Electro Ohmic Quasi-Static” finite element method (FEM) solver, which is suitable because the length-scales are much smaller than the wavelength and ohmic currents dominate over displacement currents. The simulations were run primarily to estimate spatial distribution of the effectively stimulated area, as the software is designed to simulate the induced E-field for the direct current only, without considering the temporal characteristics of tRNS. The model geometry encompassed the head and neck of the MIDA and was discretized using a grid with a resolution of 0.5–0.75 mm, a resolution found to be sufficient for the investigation-of-interest in a grid convergence analysis. The refinement was the finest near the electrodes. To account for the considerable thinness of the retina, a separate convergence analysis was conducted, which concluded that a refinement of 0.3 mm is needed to ensure accurate total field estimations in the retina. Following the construction of the final grid, which contained >109 MCells, two EM simulations were executed for each electrode pair, by assigning Dirichlet (voltage) boundary conditions of +1 V to the Oz electrode (respectively, F9 for the retina exposure configuration) and −1 V to Cz (respectively, F10). After computing the EM simulations, the resulting E-fields were normalized to the minimum, medium and maximum amplitude of the experimentally applied current intensities: 0.75, 1, and 1.5 mA for V1 stimulation (Oz and Cz pair) and 0.1, 0.2, and 0.3 mA for retina stimulation (F9 and F10 pair).

### Statistical analysis

All the statistical analyses were preregistered and did not deviate from the original plan. Statistical analyses were performed in IBM SPSS Statistics version 26.0 (IBM Corp.). All data were tested for normal distribution using the Shapiro–Wilks test. Variance is reported as SD in the main text and as SE in the figures.

First, we tested whether baseline VCT in the no tRNS condition differed across the three experimental sessions using a Bayesian rmANOVA with the factor *time* (blocks 1–2 in sessions 1–3, i.e., six consecutive time points) using the Bayes factor testing for evaluation the absence versus presence of an effect.

For all rmANOVA models, sphericity was assessed with Mauchly’s sphericity test. The threshold for statistical significance was set at α = 0.05. Bonferroni correction for multiple comparisons was applied where appropriate (i.e., *post hoc* tests; preplanned comparisons of stimulation with low, medium, and high tRNS intensity vs no tRNS baseline). Partial η^2^ (small $\eta_{p}^{2}$ = 0.01, medium $\eta_{p}^{2}$ = 0.06, large $\eta_{p}^{2}$ = 0.14; Lakens, 2013) or Cohen’s *d* (small *d* = 0.20–0.49, medium *d* = 0.50–0.80, large *d* > 0.80; Cohen, 1988) values are reported as a measure of effect-sizes.

VCT data collected in the V1 session (tRNS_V1_) was analyzed with a rmANOVA with the factor *tRNS* (no, 0.75, 1, and 1.5 mA tRNS) and the factor *block* (first, second). For each individual and each block, we determined the maximal behavioral improvement, i.e., lowest VCT measured when tRNS was applied, and the associated “optimal” tRNS intensity (ind-tRNS_V1_). The maximal behavioral improvements in the first and the second block were compared using a *t* test (two-tailed) for dependent measurements. We further tested whether ind-tRNS_V1_ intensities of the first and second block were correlated using Spearman’s rank correlation coefficient (because of categorical characteristics of ind-tRNS_V1_ intensity variable). Importantly, we determined ind-tRNS_V1_ in the first block, and then used the VCT data of the separate second block to test whether the associated VCT is lower compared with the no tRNS condition using *t* tests for dependent measures. Since we had the directional hypothesis that VCT is lower for the optimal tRNS intensity compared with no tRNS this test was one-tailed. Determining ind-tRNS_V1_ and testing its effect on VCT in two separate datasets is important to not overestimate the effect of tRNS on visual detection behavior (van der Groen and Wenderoth, 2016).

VCT data collected in the Retina session (tRNS_retina_) was analyzed with a rmANOVA with the factor of *tRNS* (no, 0.1, 0.2, and 0.3 mA tRNS) and the factor *block* (first, second). Again, for each individual and each block, we determined the maximal behavioral improvement and the associated ind-tRNS_retina_. We compared results obtained in the first and second block using the same statistical tests as for the V1 session. The maximal behavioral improvements were compared using a *t* test (two-tailed) for dependent measurements. Correlation of ind-tRNS_retina_ intensity of the first and second block was tested using Spearman’s rank correlation coefficient. We examined whether the ind-tRNS_retina_ determined based on the best behavioral performance in first block, caused VCT to be lower compared with the no tRNS condition when retested on the independent dataset (second block) using *t* tests (one-tailed) for dependent measures.

VCT data collected in the V1+Retina session (tRNS_V1+retina_) was analyzed with a rmANOVA with the factor *tRNS site* (ind-tRNS_V1,_ ind-tRNS_retina,_ ind-tRNS_V1+retina_, and no tRNS) and the factor *block* (first, second). Moreover, we compared behavioral improvement for ind-tRNS_V1_ and ind-tRNS_retina_ between sessions (tRNS_V1_ and tRNS_V1+retina_, tRNS_retina_ and tRNS_V1+retina_, respectively) using a Pearson correlation coefficient.

As a control analysis we repeated the main analyses of VCT (rmANOVA were we observed tRNS-induced significant difference) with adding cutaneous sensation as covariate (mean HR; see above, tRNS characteristics).

## Results

**Figure 4.**
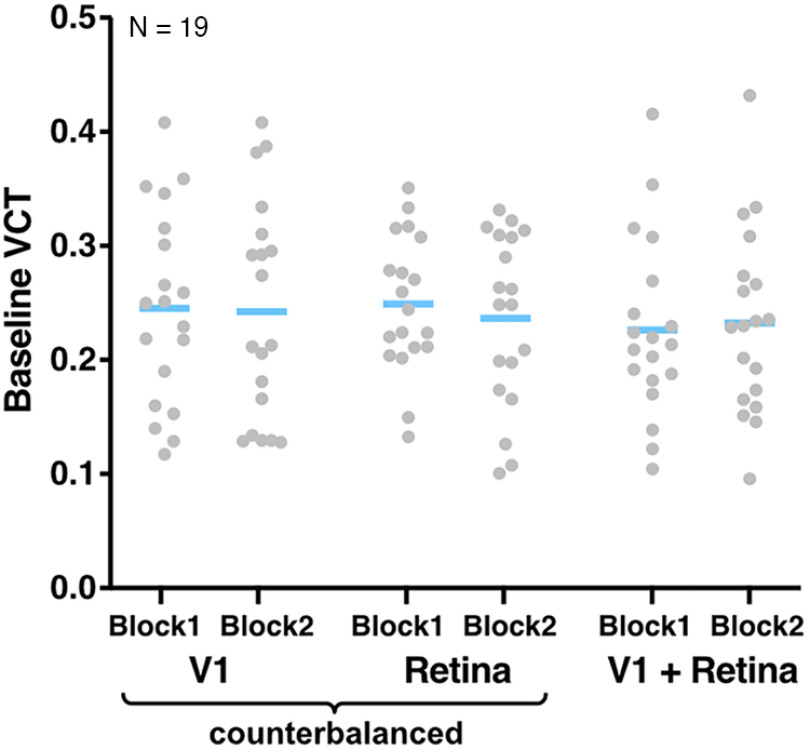
Baseline VCT measured in the no tRNS condition in both blocks in V1, Retina, and V1+Retina sessions. Blue lines indicate mean, gray dots indicate single subject data.

We first tested whether VCT measured during the no tRNS condition differed between the experimental sessions or blocks (i.e., six consecutive time points; see Fig. 4). Bayesian rmANOVA with the factor *time* (1–6) revealed that the baseline VCT measured in the no tRNS condition did not differ over time (BF_10_ = 0.06, i.e., strong evidence for the H_0_) indicating that detection performance was rather stable across sessions.

### tRNS over V1 modulates visual contrast threshold

**Figure 5.**
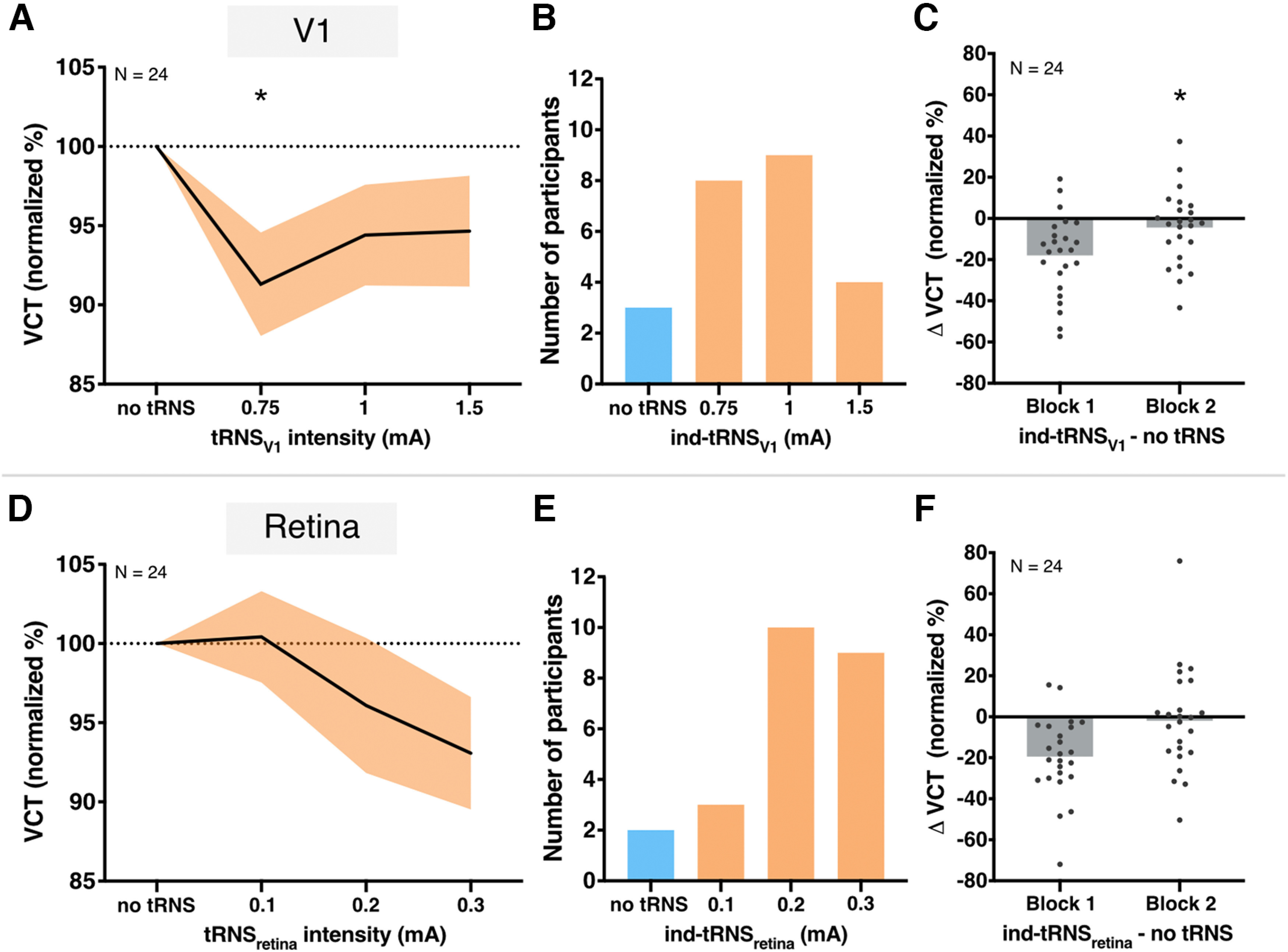
Results of V1 and Retina sessions. ***A***, Effect of tRNS_V1_ on VCT on a group level measured across first and second block in V1 session. VCT in tRNS_V1_ conditions normalized to the no stimulation baseline. Decrease in VCT reflects improvement of visual contrast sensitivity. All data mean ± SE. ***B***, Individually defined optimal tRNS_V1_ based on behavioral performance during the first block. ***C***, Detection improvement effects of individualized tRNS_V1_ in the first and second block. VCT in ind-tRNS_V1_ normalized to the no stimulation baseline. ***D***, Effect of tRNS_retina_ on VCT on a group level measured across first and second block in Retina session. VCT in tRNS_V1_ conditions normalized to the no stimulation baseline. All data mean ± SE. ***E***, Individually defined optimal tRNS_retina_ based on behavioral performance during the first block. ***F***, Detection modulation during individualized tRNS_retina_ in the first and second block. VCT in ind-tRNS_retina_ normalized to the no stimulation baseline. Gray dots indicate single subject data, gray bars indicate group mean; *p < 0.05.

In the V1 session, we investigated whether tRNS modulates the visual contrast detection when applied to V1. We measured VCT during tRNS_V1_ at intensities of 0.75, 1, to 1.5 mA versus no tRNS control condition. We found a general decrease in VCT when tRNS was applied (*tRNS* main effect: *F*_(3,69)_ = 4.54, *p* = 0.006, $\eta_{p}^{2}$ = 0.165) indicating that adding noise to V1 improved contrast sensitivity (Fig. 5*A*). *Post hoc* comparisons revealed that the 0.75 mA stimulation was most effective in boosting contrast processing, which differed significantly from the no tRNS control condition (*p* = 0.022, mean difference, MD = −8.69 ± 15.99%). There was also a trend toward significantly lower VCT during 1 mA stimulation (*p* = 0.06, MD = −5.6 ± 15.63%). Neither the main effect of *block* (*F*_(1,23)_ = 0.18, *p* = 0.678) nor tRNS*block interaction (*F*_(3,69)_ = 0.82, *p* = 0.488) reached significance.

The control measurement of cutaneous sensation revealed that most of our participants could detect tRNS_V1_ conditions (HR at 0.75 mA = 63.54 ± 31.26%, 1 mA = 73.96 ± 30.82%, 1.5 mA = 90.63 ± 23.09%, mean HR = 76.04 ± 22.16%). We reanalyzed our main outcome parameter by adding sensation detection HR as a covariate. The main effect of *tRNS* remained highly significant (*F*_(3,66)_ = 4.17, *p* = 0.009, $\eta_{p}^{2}$ = 0.159), making it unlikely that cutaneous sensation was the main driver of our results.

When comparing tRNS-induced effects between the first and second block we found that the maximal behavioral improvement (i.e., the maximal tRNS_V1_-induced lowering of the VCT relative to the no tRNS condition) did not differ between the first (MD = −17.98 ± 19.6%) and the second block (MD = −16.63 ± 15.11%, *t*_(23)_ = −0.304, *p* = 0.764). However, participants’ optimal ind-tRNS_V1_ intensity of block 1 and 2 (i.e., the tRNS intensity causing the largest VCT reduction in each block) were not correlated (ρ = 0.225, *p* = 0.290). This suggests that participants may have profited to a different extend from variable intensities between the blocks.

Finally, we determined ind-tRNS_V1_ in the first block (Fig. 5*B*) and tested whether the selected intensity caused a decrease in VCT compared with the no tRNS condition using the data of the second block. Note, that Figure 5*B* shows the distribution of conditions in which participants performed the best (including three participants with the best performance in the no tRNS condition, blue bar). The ind-tRNS_V1_ was always selected from the conditions where tRNS_V1_ was applied (see Materials and Methods). Accordingly, Figure 5*C* shows some values > 0 in first block indicating that some participants did not benefit from the stimulation. Indeed, in the second block VCT decreased in ind-tRNS_V1_ relative to the no tRNS baseline in 15 out of 24 individuals (MD = −4.45 ± 17.9%) and this effect reached statistical significance (*t*_(23)_ = 1.72, *p* = 0.049, *d* = 0.2; Fig. 5*C*, second block). Importantly, the optimal ind-tRNS_V1_ intensity and the associated VCT effect were determined on independent datasets to avoid circularity.

### tRNS over the retina does not modulate visual contrast threshold consistently

In the Retina session, we explored the effects of tRNS applied over the retina on visual contrast detection. VCT was measured during tRNS_retina_ at intensities of 0.1, 0.2, to 0.3 mA versus no tRNS control condition. Although, on the group level, we observed decrease in VCT with increasing tRNS_retina_ intensity (MD = −6.93 ± 17.39% on average in the first and second block for 0.3 mA) the effect was not significant (*F*_(3,69)_ = 1.69, *p* = 0.177; Fig. 5*D*). There was also no main effect of *block* (*F*_(1,23)_ = 0.04, *p* = 0.840) or tRNS*block interaction (*F*_(3,69)_ = 0.55, *p* = 0.652). The maximal behavioral improvements, defined as the maximal tRNS_retina_-induced lowering of the VCT were not significantly different between the first (MD = −19.44 ± 19.43%) and the second (MD = −11.96 ± 22.79%) block (*t*_(23)_ = −1.197, *p* = 0.243). The optimal ind-tRNS_retina_ intensity defined in the first and second block were not significantly correlated among participants (ρ = 0.321, *p* = 0.126), indicating that variable intensities between the blocks did not consistently affect processing in the retina. The ind-tRNS_retina_ determined in the first block (Fig. 5*E*; note that two participants showed their best performance in the no tRNS condition, blue bar) did not significantly lower the VCT compared with the no tRNS condition when retested on the independent VCT dataset of block 2 (*t*_(23)_ = 1.05, *p* = 0.15, VCT decrease in 13 out of 24 individuals, MD = −1.89 ± 25.29%; Fig. 5*F*).

Similarly to the tRNS_V1_ session, the control measurement of cutaneous sensation revealed that the participants could detect tRNS_retina_ conditions (HR at 0.1 mA = 18.75 ± 25.8%, 0.2 mA = 27.08 ± 31.2%, 0.3 mA = 37.5 ± 39.01%, mean HR = 24.11 ± 27.34%). As we did not observe a significant effect of tRNS_retina_, here we did not run an additional control analysis.

### No effects of simultaneous tRNS of V1 and retina on visual contrast threshold

**Figure 6.**
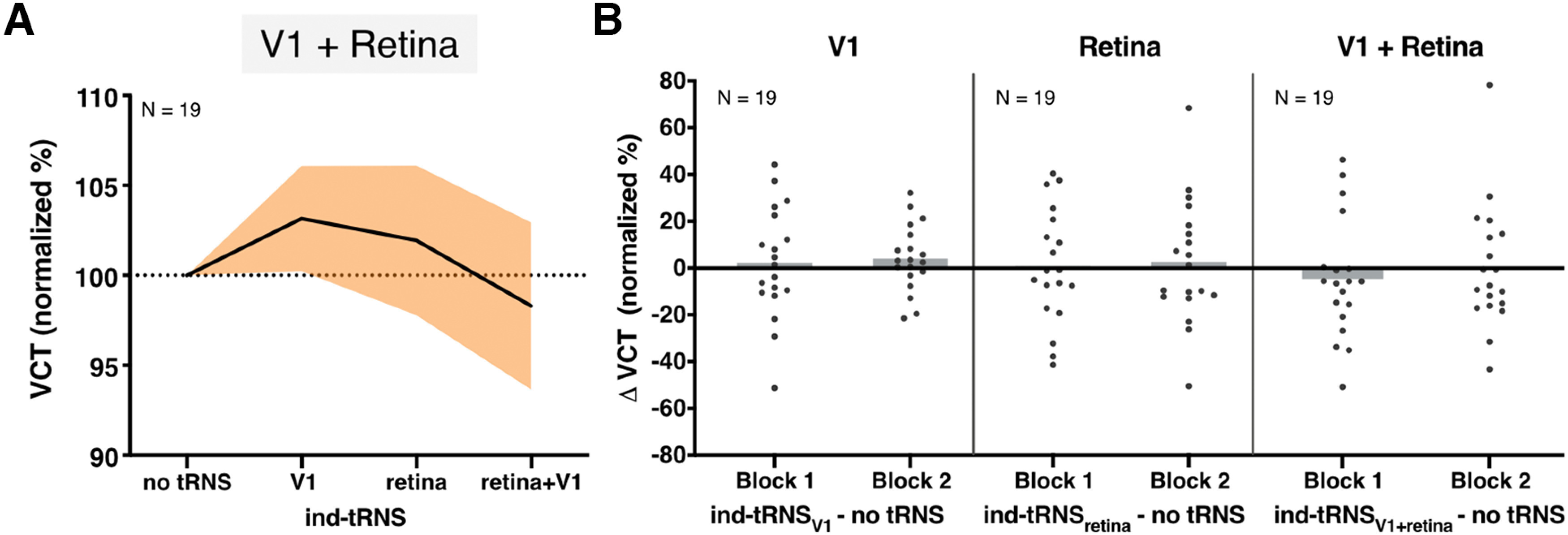
Results of V1+Retina session. ***A***, Effect of individualized tRNS_V1_, tRNS_retina_, and tRNS_V1+retina_ on VCT on a group level measured across blocks 1 and 2 in V1+Retina session. All data mean ± SE. ***B***, The detection modulation during participants’ optimal ind-tRNS_V1_, ind-tRNS_retina_, and simultaneous ind-tRNS_V1+retina_ in blocks 1 and 2 of V1+Retina session. Gray dots indicate single subject data, gray bars indicate group mean.

The aim of V1+Retina session was to explore whether the effects of ind-tRNS_V1_ and ind-tRNS_retina_ determined in sessions 1 and 2 would have additive effects when combined during simultaneous V1 and retina stimulation (Fig. 6*A*). Against our hypothesis, we did not observe a consistent decrease in VCT on the group level, neither when considering *tRNS site* (*F*_(3,54)_ = 0.54, *p* = 0.660), *block* (*F*_(1,18)_ = 2.73, *p* = 0.116) nor tRNS site*block interaction (*F*_(3,54)_ = 0.31, *p* = 0.822). Although the simultaneous stimulation with ind-tRNS_V1+retina_ led to a decrease in VCT in the first block (MD = −4.12 ± 25.64%), this difference was not significant (*t*_(18)_ = 0.83, *p* = 0.21; Fig. 6*B*).

**Figure 7.**
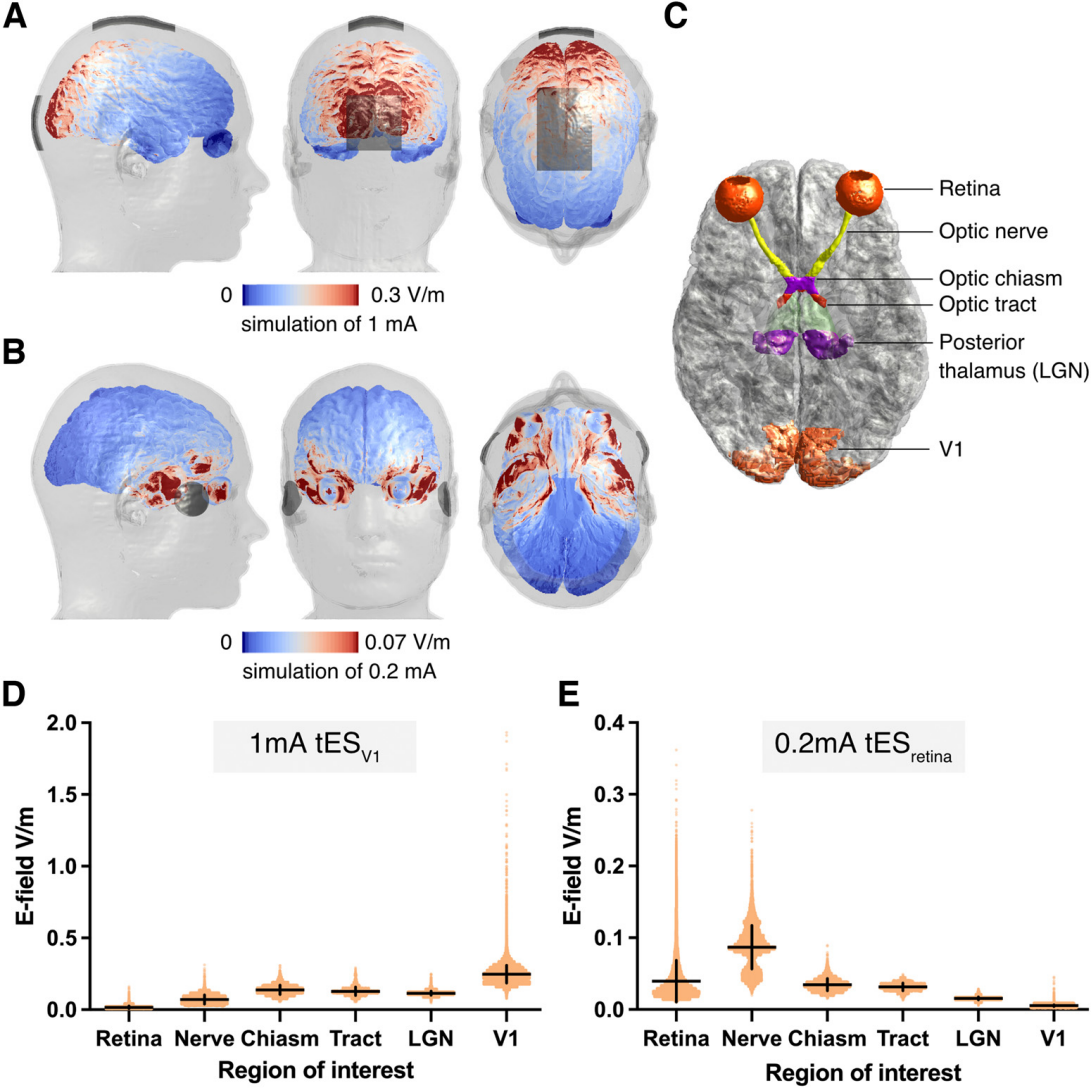
Results of E-field modeling. ***A***, Distribution of induced E-field during 1-mA tES targeting V1. Gray patches represent electrodes montage. ***B***, Distribution of induced E-field during 0.2-mA tES targeting the retina. Gray patches represent electrodes montage. ***C***, Regions of interest included in E-field modeling. ***D***, Distribution of induced E-field within six regions of interest during 1-mA tES targeting V1. Orange dots represent single voxels within masks, horizontal and vertical lines represent mean and SD, respectively. ***E***, Distribution of induced E-field within six regions of interest during 0.2-mA tES targeting the retina. Orange dots represent single voxels within masks, horizontal and vertical lines represent mean and SD, respectively.

The control measurement of cutaneous sensation in the third session revealed that the participants could detect tRNS conditions (HR at ind-tRNS_V1_ = 56.58 ± 43.97%, ind-tRNS_retina_ = 19.74 ± 31.82%, ind-tRNS_V1+retina_ = 67.11 ± 39.13%, mean HR = 47.81 ± 28.03%). As we did not observe a significant effect of the stimulation, we did not run an additional control analysis.

In the third session we also retested the effects of individually optimized tRNS intensities defined in V1 and Retina sessions. The effect of ind-tRNS_V1_ found in V1 session was not reproduced between sessions when VCT was measured during ind-tRNS_V1_ in session 3 (*t*_(18)_ = −0.18, *p* = 0.43, MD = 2.24 ± 23.63%, and *t*_(18)_ = −1.37, *p* = 0.09, MD = 4.1 ± 14.28%; Fig. 6*B*, first and second blocks, respectively). There was also no association between behavioral improvements measured during ind-tRNS_V1_ in the first blocks of V1 and V1+Retina sessions (*r* = 0.12, *p* = 0.961, *N* = 19), indicating that once-optimized tRNS intensity does not lead to consistent effects between sessions. Similarly to Retina session, participants’ ind-tRNS_retina_ did not lower the VCT compared with the no tRNS condition when retested on the VCT data in session 3 (*t*_(18)_ = 0.12, *p* = 0.45, MD = 1.02 ± 24.57%, and *t*_(18)_ = −0.17, *p* = 0.44, MD = 2.91 ± 26.51%; Fig. 6*B*, first and second blocks, respectively). There was also no association between behavioral improvements measured during ind-tRNS_retina_ in the first blocks of Retina and V1+Retina sessions (*r* = −0.252, *p* = 0.297, *N* = 19).

### E-field predictions during stimulation of the visual system

To characterize the exposure of the two stimulation conditions, namely, V1 and the retina stimulation, for different current intensities (0.75, 1, and 1.5 mA for V1 stimulation and 0.1, 0.2, and 0.3 mA for retina stimulation), we compared the mean and standard deviation (SD) of the total E-field in six regions along the retino-cortical pathway, namely the retina, optic nerve, optic chiasm, optic tract, posterior thalamus (including LGN), and V1 area. The results are presented in Table 1 (for V1 montage) and Table 2 (for Retina montage).

**Table 1:** E-field induced along the retino-cortical pathway during tES targeting V1 (electrodes configuration Oz and Cz)

tES targeting V1			
Mask	E-field 0.75 mA	E-field 1 mA	E-field 1.5 mA
Retina	0.01 ± 0.01	0.02 ± 0.01	0.02 ± 0.01
Optic nerve	0.05 ± 0.02	0.07 ± 0.03	0.11 ± 0.05
Optic chiasm	0.1 ± 0.02	0.14 ± 0.03	0.21 ± 0.05
Optic tract	0.1 ± 0.02	0.13 ± 0.03	0.19 ± 0.04
LGN	0.09 ± 0.01	0.11 ± 0.01	0.17 ± 0.02
V1	0.19 ± 0.05	0.25 ± 0.06	0.37 ± 0.09

E-field values represent V/m, mean ± SD.

**Table 2:** E-field induced along the retino-cortical pathway during tES targeting the retina (electrodes configuration F9 and F10)

tES targeting retina			
Mask	E-field 0.1 mA	E-field 0.2 mA	E-field 0.3 mA
Retina	0.02 ± 0.01	0.04 ± 0.03	0.06 ± 0.04
Optic nerve	0.04 ± 0.02	0.09 ± 0.03	0.13 ± 0.05
Optic chiasm	0.02 ± 0	0.03 ± 0.01	0.05 ± 0.01
Optic tract	0.02 ± 0	0.03 ± 0.01	0.05 ± 0.01
LGN	0.01 ± 0	0.02 ± 0	0.02 ± 0
V1	0 ± 0	0.01 ± 0	0.01 ± 0

E-field values represent V/m, mean ± SD.

According to the simulation predictions, Oz and Cz configuration efficiently targeted the V1 region (0.25 ± 0.06 V/m) and led to average stimulation of 0.016 ± 0.01 in the retina for the medium (1 mA) current intensity (Fig. 7*A*,*D*). By comparison, the medium V1 exposure with the F9 and F10 configuration was lower, namely 0.2 mA, but it produced a larger field (0.04 ± 0.03 V/m) in the retina than the previous configuration, making it more suitable for targeting the retina (Fig. 7*B*,*E*). Still, the predicted retinal E-field is considerably lower than that required to evoke significant neural activity, according to a recent meta-analysis, which identified a threshold of 0.2 V/m for neural entrainment (Alekseichuk et al., 2022). To reach this threshold, or a level of retinal exposure similar to that of the V1 region under the Oz and Cz configuration, a stimulation intensity of 1.25 mA on the F9 and F10 electrodes would be required, which is likely to be painful and disturbing for participants. Interestingly, the simulation of the F9 and F10 configuration predicted higher total E-field in the region of the optic nerve (0.09 ± 0.03) than in the retina (Fig. 7*E*). However, a similar optic nerve exposure strength was also found for the Oz and Cz pair (0.07 ± 0.03; Fig. 7*D*) without triggering visual effects, it can be assumed that the observed effects for the F9 and F10 montage were primarily driven by stimulation of retina and V1, rather than the optic nerve.

## Discussion

The present study investigated the effects of tRNS on visual contrast sensitivity, when applied to different neuronal substrates along the retino-cortical pathway. We measured VCT during tRNS_V1_ and tRNS_retina_ and tRNS_V1+retina_ across three experimental sessions. We found consistent tRNS-induced enhancement of visual contrast detection during V1 stimulation (Fig. 5*A–C*) but not during retina stimulation (Fig. 5*D–F*). We also did not observe any additive effects on contrast detection when noise stimulation was simultaneously applied to V1 and retina (Fig. 6*A*,*B*). The online modulation effects of individually optimized tRNS_V1_ intensities were replicated within session (i.e., across two separate blocks; Fig. 5*C*), but not between experimental sessions (Fig. 6*B*). Our findings likely reflect acute effects on contrast processing rather than after-effects, as stimulation was only applied for short intervals (2 s) and always interleaved with control (no tRNS) conditions.

### tRNS improves visual sensitivity in V1

Our findings confirm previous evidence that the detection of visual stimuli is immediately enhanced when tRNS is added centrally to V1 at optimal intensity (Fig. 5*A*; van der Groen and Wenderoth, 2016), although a different outcome measurement was used (i.e., VCT instead of detection accuracy of subthreshold stimuli). As such, it constitutes to a conceptual replication of the earlier study. The modulation observed here was characterized by large effect size ( $\eta_{p}^{2}$ = 0.165; Fig. 5*A*), stronger than the intermediate effect size (Cohen’s *d* = 0.77) found by van der Groen and Wenderoth (2016). Thus, the threshold tracking procedure (Watson and Pelli, 1983; Brainard, 1997; Pelli, 1997; Kleiner et al., 2007) used in our experiments seems to provide a sensitive and reliable estimate of behavioral effects of tRNS_V1_. Moreover, the 4-AFC task protocol used in our study was shown to be more efficient for threshold estimation than commonly used 2-AFC (Jäkel and Wichmann, 2006).

It has been argued previously that tRNS benefits visual detection via SR mechanism, i.e., the detection probability of weak, subthreshold signals in nonlinear systems can be enhanced if optimally adjusted random noise is added (Moss et al., 2004; McDonnell and Abbott, 2009). One indicative feature of the SR phenomenon is an “inverted U-shape” dose–response relationship between the noise intensity and exhibited noise benefits, i.e., while the optimal level of noise benefits performance, excessive noise is detrimental (van der Groen and Wenderoth, 2016; van der Groen et al., 2018; Pavan et al., 2019). In the V1 session, we could show that task performance accuracy changed according to an “inverted-U-shape” function with increasing tRNS_V1_ intensities (ranging from 0 to 1.5 mA) which is consistent with a SR mechanism. Note, that enhancement in visual detection performance was reflected in decreased VCT in relation to the no stimulation baseline (i.e., “U-shape” dose-threshold relationship here; Fig. 5*A*). This improvement was most likely driven by effective stimulation of V1 rather than unspecific tRNS effects such as cutaneous stimulation and associated effects on arousal, as confirmed in the additional analysis using cutaneous sensation detection during tRNS as a covariate.

Based on the behavioral task performance, we determined which tRNS_V1_ intensity was optimal on the individual level (i.e., ind-tRNS_V1_ causing the lowest VCT for each participant; Fig. 5*B*). The optimal noise intensities varied across individuals, similar to effects previously shown for noise added both to the stimulus (Collins et al., 1996; Martínez et al., 2007) or centrally to V1 (van der Groen and Wenderoth, 2016). The ind-tRNS_V1_ intensities defined separately in first and second blocks of V1 session were not correlated, suggesting that the intensities leading to the maximal improvement effects within participants were not always the same between the measurements. Importantly, we demonstrated that the ind-tRNS_V1_ (from first block) results in consistent online enhancement effects when retested on the independent data set (VCT in second block) within the experimental session (Fig. 5*C*). This indicates that an individually optimized tRNS_V1_ intensity can be considered stable and effective when applied across multiple blocks of a measurement. Notably, the effect of ind-tRNS_V1_ was not replicable on different session (Fig. 6*B*; see below, Intersession variability in the effects of individualized tRNS protocol on contrast sensitivity).

The modeling showed that E-field induced in V1 during stimulation was around 0.19V/m to 0.37V/m, which confirms that the area was stimulated strong enough to modulate visual processing (Alekseichuk et al., 2022).

Our study contributes to the evidence for SR as a mechanism underlying online visual processing modulation when tRNS is applied to neural networks in human cortex (van der Groen and Wenderoth, 2016; van der Groen et al., 2018, 2019; Battaglini et al., 2019, 2020; Pavan et al., 2019; see review, see Potok et al., 2022)

### Inconsistencies in the effects of noise on retinal processing of contrast

The present study did not demonstrate systematic noise benefits at the level of the retina. Thus, suggesting that previously reported SR effects on contrast detection might derive mainly from cortical rather than retinal processing. It also shows that SR effects might differ based on the specific characteristic of the stimulated neural tissue.

In our study, we targeted the retina bilaterally with tRNS, to investigate its effects on contrast sensitivity. Although increases in tRNS_retina_ intensity resulted in decreases in VCT, reflecting relative task performance improvements (Fig. 5*D*), the effects did not reach statistical significance. Similarly to tRNS_V1_, the effects of tRNS_retina_ were variable across study participants. However, even individually determined optimal intensities of tRNS_retina_ did not result in consistent visual processing improvements when retested in separate blocks, both within and between sessions (Fig. 5*F*, Fig. 6*B*).

Why did tRNS improve contrast detection when applied to V1 but not when applied to retina? In contrast to V1, the retina is characterized by much larger temporal frequency bandwidth toward which it is responsive. One study measured cat ganglion cell responsivity toward temporal frequencies ranging from 0.1 to 100 Hz (Frishman et al., 1987). Further studies have shown a similar range of temporal frequency bandwidth in monkey retina (Benardete and Kaplan, 1999) and even higher cutoff frequencies in response to electrical stimulation in rabbit retina (Cai et al., 2011). Moreover, a fMRI study in humans showed a much higher temporal frequency bandwidth cutoff in human LGN (recipient of retinal ganglion cells’ signals) compared with human V1 (Bayram et al., 2016), where the strongest effects are observed for narrow bandwidth of around 4–8 Hz (Fawcett et al., 2004). Taken together, stimulus processing at the level of the retina seems to cover a much wider range of temporal frequencies than in V1 and to be more variable. Thus, it is possible that the range of tRNS frequencies used in our experiments, i.e., 100–640 Hz might have been too close to the intrinsic signaling frequencies in the retinal circuitry and in ganglion cells to induce the typical SR effect. V1 neurons, by contrast, respond to frequencies which are one to two magnitudes lower than the tRNS frequencies; and therefore, larger noise benefits could be observed.

Alternatively, the weak effects of tRNS_retina_ might simply be because of filtering properties of retinal neurons. A recent study used amplitude modulated tACS (AM-tACS) applied to the retina to investigate the efficacy of different carrier frequencies to induce phosphenes. AM-tACS waveforms comprised of different carrier (50, 200, 1000 Hz) and modulation frequencies (8, 16, 28 Hz). They found that from the conditions using different carrier frequencies only the lowest one was able to induce phosphenes (Thiele et al., 2021). Thus, suggesting the low-pass nature of retinal neurons which greatly reduces the stimulation effectiveness of evoking suprathreshold response (Deans et al., 2007; Thiele et al., 2021). The researchers point out, however, that their findings do not rule out potential subthreshold modulations of neural activity during AM-tACS with high carrier frequencies.

In the Retina session we observed gradual decrease in VCT in comparison to the baseline with increasing tRNS_retina_ intensity on the group level (Fig. 5*D*). Although this effect was not significant, we cannot exclude that VCT would decrease further when higher tRNS_retina_ intensities were used. This limits the interpretation of the negative results during tRNS_retina_, and the direct comparisons between tRNS_retina_ and tRNS_V1_.

The modeling showed that the induced E-field varied from 0.02V/m to 0.06V/m in the retina and from 0.04V/m to 0.13V/m in the optic nerve during stimulation applied when targeting the retina. These mean values are most probably too low to modulate neuronal processing (Alekseichuk et al., 2022). However, the minimal gradual decrease in contrast processing might be related to the fact that some parts (here voxels) of the stimulated area might have been stimulated to a greater degree according to the modeling (see the range of values, up to >0.2V/m, in Fig. 7*E*).

We have based our stimulation intensities on studies using repetitive transorbital alternating current stimulation with similar intensities (Gall et al., 2010, 2011; Fedorov et al., 2011) and demonstrated improved vision in patients with damaged optic nerve (see also Sabel et al., 2020). Similarly, our pilot experiment measuring flickering threshold for low-frequency tRNS targeting retina revealed that the stimulation intensity of around 0.146 mA was strong enough to reach the retina and induce flickering sensations. However, it is possible that the induced current is more strongly attenuated in our study (which used much higher stimulation frequencies) because of the filter properties of retinal neurons. Moreover, in the aforementioned studies, the alternating current was delivered using set of four electrodes positioned above and below participants’ eyes. Such electrodes placement results in different direction of the current and related orientation of the induced electric field than bilateral placement used in this study (Fig. 2*A*). Thus, electrodes montage used here might have been suboptimal for retinal ganglion cell stimulation (Dmochowski et al., 2012; Lee et al., 2021). Finally, the E-field modeling showed that the optic nerve was in fact stimulated stronger than the retina itself under our experimental conditions. It is then possible that the flickering effects observed in our pilot experiment were caused by stimulation of the optic nerve and not the retina.

There are several limitations of this modeling study that should be addressed in future research. Specifically, further investigation of the relevant exposure component of the predicted E-fields is necessary, as the current work only considered the absolute field magnitude. Future studies should provide information on the normal component in cortical regions, as well as the activating function along the optic nerve fibers. These additional analyses would provide more comprehensive insights into the impact of the two stimulation setups on the visual system.

Future studies could investigate the influence of hf-tRNS on the retina using different electrodes montage or higher stimulation intensity, preferably matching the electric field induced in the V1 (here estimated as 1.25mA, which might be unpleasant for participants). Note, however, that the characteristics of the current waveform (variable intensities and frequencies) might be challenging for reliable simulation of the E-field induced by tRNS. Additionally, special attention should be drawn to the feasibility and safety when higher stimulation intensities are delivered over the temples, as the skin around the eyes might be sensitive.

In summary, we found no evidence that tRNS affects contrast detection at the retinal level. This is interesting from a methodological perspective since it may rule out that applying tRNS over V1 elicits confounding effects in the retina, as previously discussed for tACS experiments (Schutter and Hortensius, 2010; Schutter, 2016).

### Intersession variability in the effects of individualized tRNS protocol on contrast sensitivity

The influence of individually optimized tRNS on VCT, defined separately for both V1 and the retina in experimental sessions 1 and 2, were retested in session 3. The effects of neither ind-tRNS_V1_, nor ind-tRNS_retina_ were replicated (Fig. 6*A*,*B*), indicating that optimal tRNS intensity for maximum task performance improvement needs to be individually re-adjusted on each experimental session. This might be of particular importance when tRNS is used for clinical purpose, such as in combination with perceptual learning for improving visual functions (Camilleri et al., 2014, 2016; Moret et al., 2018; Herpich et al., 2019; Donkor et al., 2021). These results confirm the well-known variability in the effectiveness of noninvasive brain stimulation (Polanía et al., 2018) and the necessity of carefully designing optimal protocols (Bergmann et al., 2016; Bergmann and Hartwigsen, 2020). The differences in effectiveness of preselected tRNS intensities could result from intrinsic factors such as the participants’ arousal levels or attentional states. Additionally, although we made sure that our procedure was well standardized, there might have been slight differences in the precise electrodes montage or amount of electroconductive gel, potentially resulting in variability of the electric field induced by tRNS of selected intensity across sessions (Polanía et al., 2018).

Importantly, the overall design of the third session (V1+Retina) was different from the previous two regarding the stimulation conditions. Here, the preselected stimulation conditions of ind-tRNS_V1_, ind-tRNS_retina_, and ind-tRNS_V1+retina_ were randomly interleaved. Although we expected to induce only acute effects, it is possible that (1) introducing noise at different levels of the visual system in short time breaks was not beneficial for the task performance, or (2) using a range of tRNS intensities, as in the first and second session might have increased the probability of acute noise benefits in comparison to stimulation with only one preselected intensity.

It is also worth noting that the substantial delay between V1/Retina sessions, and V1+Retina session (five months on average) because of the COVID-19 pandemic (Bikson et al., 2020) could have also influenced this variability. As the modulation of VCT with ind-tRNS_V1_ or ind-tRNS_retina_ was not replicated in session 3, it is not possible to draw conclusions about the simultaneous effect of V1 and the retina stimulation (Fig. 6*A*,*B*).

In conclusion, our study confirms previous findings that tRNS might enhance visual signal processing of cortical networks via the SR mechanism (van der Groen and Wenderoth, 2016; Potok et al., 2021). When probing the effects of tRNS on contrast sensitivity along the retino-cortical pathway, we demonstrated that visual processing in V1 benefits from tRNS-induced modulation. Stimulation of the retina did not lead to significant improvements in contrast detection. As the stimulation conditions varied between the two areas, and an increased stimulation intensity might potentially affect the retinal processing in a different manner, we cannot directly compare and interpret the effects between tRNS_V1_ and tRNS_retina_. Finally, we found that the individual optimal tRNS intensity applied to V1 to enhance contract detection appears to vary across sessions. The appropriate adjustment of optimal tRNS intensity is therefore important to consider when designing tRNS protocols for perceptual enhancement.

## Synthesis

Reviewing Editor: Nicholas J. Priebe, University of Texas at Austin Decisions are customarily a result of the Reviewing Editor and the peer reviewers coming together and discussing their recommendations until a consensus is reached. When revisions are invited, a fact-based synthesis statement explaining their decision and outlining what is needed to prepare a revision will be listed below. The following reviewer(s) agreed to reveal their identity: NONE.

The reviewers all agree that the manuscript has been revised appropriately.
